# Relationship between kidney function and healthy life expectancy: A historical cohort study

**DOI:** 10.1186/s12882-024-03843-0

**Published:** 2025-01-13

**Authors:** Hisayuki Ogura, Tadashi Toyama, Hikaru Samuta, Kohei Hirako, Tomoya Itatani, Shiori Nakagawa, Megumi Oshima, Shinji Kitajima, Akinori Hara, Norihiko Sakai, Miho Shimizu, Tomoyuki Takura, Takashi Wada, Yasunori Iwata

**Affiliations:** 1 Department of Nephrology and Rheumatology, Graduate School of Medical Sciences, Kanazawa University, Ishikawa, 920-8641 Japan; 2 Department of Nephrology, Faculty of Medical Sciences, University of Fukui, Fukui, 910-1193 Japan; 3 Faculty of Transdisciplinary Sciences for Innovation, Institute of Transdisciplinary Sciences for Innovation, Kanazawa University, Ishikawa, 920-1192 Japan; 4 Faculty of Interdisciplinary Economics Department of Interdisciplinary Economics, Kinjo University, Ishikawa, 924-8511 Japan; 5 School of Nursing, Faculty of Medicine, University of Miyazaki, Miyazaki, 889-1692 Japan; 6 Department of Hygiene and Public Health, Graduate School of Medical Sciences, Kanazawa University, Ishikawa, 920-8641 Japan; 7 Department of Healthcare Economics and Health Policy, Graduate School of Medicine, The University of Tokyo, Tokyo, 113-8655 Japan; 8 Department of Health Care Services Management, Nihon University School of Medicine, Tokyo, 173-8610 Japan

**Keywords:** Cohort study, Healthy life expectancy, Kidney function, Long-term care costs, Medical care costs

## Abstract

**Background:**

The impact of chronic kidney disease (CKD) on healthy life expectancy and healthcare costs requires research. This study examined associations between CKD and healthy life expectancy, and its economic burden.

**Methods:**

This study of community-dwelling adults residing in Hakui City, Ishikawa Prefecture, Japan used data from the National Health Insurance database between 2012 and 2022. Participants were grouped by baseline estimated glomerular filtration rate (eGFR) (< 45, ≥ 45 to < 60, ≥60 to < 75, ≥75 to < 90, and ≥ 90 mL/min/1.73 m²). The primary endpoint was a composite of becoming a care level ≥ 2 or death. Multivariable Cox proportional hazards models were used to calculate the risk regarding time to the primary endpoint. Secondary endpoints were the annual medical and long-term care costs.

**Results:**

The 5,592 participants had a mean follow-up of 6.4 years. The hazard ratio was 1.86 (95% confidence interval [CI]: 1.35 to 2.55) for the eGFR < 45 group and 1.60 (95% CI: 1.13 to 2.25) for the eGFR ≥ 90 group, both compared with the eGFR ≥ 60 to < 75 group. Both annual costs were significantly higher in the lower eGFR groups than in the higher eGFR groups. For the eGFR < 45 group, the median medical care cost was 0.38 million yen/year in all participants and the median long-term care cost was 0.40 million yen/year in primary endpoint achievers. A lower eGFR was correlated with longer unhealthy years of life.

**Conclusions:**

Higher and lower eGFRs were associated with increased risks of reduced healthy life expectancy. A lower eGFR was associated with higher medical and long-term care costs.

**Supplementary Information:**

The online version contains supplementary material available at 10.1186/s12882-024-03843-0.

## Background

Many high-income countries are facing increasing demands for healthcare services owing to an aging population. According to estimates, 16% of the population will be over the age of 65 by 2050 [1]. This demographic shift increases the need to extend both average and healthy life expectancy. However, longer life expectancy does not always result in longer healthy life expectancy [2, 3].

Healthy life expectancy could be shortened by several factors, including cerebrovascular disease, heart disease, and lifestyle-related conditions [4–6]. Of these, chronic kidney disease (CKD) has recently gained attention, particularly in aging countries. CKD incidence tends to increase with age and is associated with several serious diseases, including cerebrovascular disease, heart disease, and dementia [7, 8]. However, few studies have reported on the relationship between CKD and healthy life expectancy [8].

Comprehensive support from both medical and long-term care services is increasingly critical in an aging society. In Japan, medical care services are provided through universal health insurance. To improve the quality of life of the older population, the Japanese government introduced public long-term care insurance in 2000, to complement the existing public medical care insurance. Although the impact of CKD on the cost of medical care has been previously reported [9–11], its impact on the cost of long-term care has not been well studied. To continue to support the older population, it is necessary to understand the economic burden of CKD from the viewpoint of both medical and long-term care costs.

This study aimed to clarify the association between CKD and healthy life expectancy and evaluate the impact of CKD on medical and long-term care costs. The results of this study could contribute to the development of healthcare strategies to improve the quality of life of older adults.

## Methods

### Ethics statements

Our study was conducted in accordance with the ethical standards postulated by the Declaration of Helsinki of 1975, as revised in 2013. This study protocol was approved by the Ethics Committee of Kanazawa University (approval number 053). The Ethics Review Board waived the requirement for written informed consent because all data were pseudonymized and provided for us by Hakui City Hall before we accessed and analyzed it. Participants were informed via our website that they could opt out from the study at any time. Participants who indicated that they could not cooperate with our study protocol were excluded from the analysis upon our request to Hakui City Hall staff, who excluded these participants using a code key from the data. Finally, the revised data were provided to us.

### Study design and participants

This historical cohort study of community-dwelling adults used data sourced from the Kokuho Database (KDB), a National Health Insurance database, and it aimed to analyze the estimated glomerular filtration rate (eGFR) as a potential prognostic factor for healthy life expectancy. The target population comprised individuals registered in the KDB in Hakui City, Ishikawa Prefecture, Japan. The inclusion criteria were: (1) age ≥ 40 years (the eligibility age for long-term care insurance), (2) enrollment in the KDB, and (3) health checkups at least once between 2012 and 2022. The exclusion criteria were: (1) no covariate baseline data and (2) care level ≥ 2 at baseline.

### Data source for analysis

The KDB contains data on individuals enrolled in Japan’s National Health Insurance, including information on the basic resident registration system, medical care, long-term care, and health checkups. These data have been collected regularly by municipalities across Japan since April 2012. We used the KDB managed by Hakui City Hall in Ishikawa Prefecture, Japan. Hakui City is a municipality with a population of approximately 23,000 in 2012.

In this analysis, dates of death were sourced from the basic resident registration system data. The costs of medical and long-term care were calculated from the respective datasets. Under Japan’s universal health insurance system, insured individuals pay 10–30% of the medical and long-term care costs, and the insurers pay the rest. Our analysis considered the total cost of the services combining the insured and insurer payments and excluding the non-covered services and transportation (Additional file 1). Levels of social support were determined using long-term care data. Data on blood pressure levels, body mass index (BMI), laboratory results, and lifestyle habits were obtained from the health checkup data.

### Assessment of healthy life expectancy

Healthy life expectancy was assessed on the basis of the Healthy Life Expectancy Calculation Guidelines published by the Japanese government [12]. Healthy life expectancy is the length of time a person lives without limitations in the activities of daily living due to health problems. An unhealthy state is a condition requiring care with a care level ≥ 2 in the long-term care insurance system in Japan (level ≥ 5 of the 8 social support levels; Additional file 2) [13]. Based on these, healthy life expectancy was defined as the time until care level ≥ 2 was required or death.

### Statistical analysis

We grouped participants into five categories according to their baseline eGFR (< 45, ≥ 45 to < 60, ≥60 to < 75, ≥75 to < 89, and ≥ 90 mL/min/1.73 m^2^). This classification was based on the Kidney Disease: Improving Global Outcomes (KDIGO) guidelines for CKD staging [14] and previous reports on mortality risk by eGFR values [15]. The eGFR used for the classification was calculated from a single creatinine measurement obtained during the participants’ health checkups in their first year of registration. Continuous variables are presented as means and standard deviations and categorical variables as proportions.

eGFR was calculated based on serum creatinine levels using the formula designed for Japanese people [16]. Blood pressure was measured in a sitting position after the participants had rested. The criteria for defining diabetes mellitus included: (1) a fasting plasma glucose ≥ 126 mg/dL (≥ 7.0 mmol/L), (2) hemoglobin A1c level ≥ 6.5%, or (3) use of antihyperglycemic agents [17].

The primary endpoint was a composite of becoming a care level ≥ 2 or death. Multivariable Cox proportional hazards models were used to calculate the risk for each eGFR group regarding time to the primary endpoint. The secondary endpoints were the annual medical and long-term care costs. The annual costs were calculated for all participants: those who did and did not achieve the primary endpoint. The annual costs for each individual were calculated as the average of the total costs divided by the total observation period, including the period after the achievement of the primary endpoint (Additional file 1). Trends in the annual costs were evaluated using linear regression analysis. In the multivariable Cox proportional hazards models, the dependent variable was the time to reach a care level ≥ 2 or death, and the independent variables included the eGFR groups. We used the eGFR ≥ 60 to < 75 group as the reference group because previous studies showed that the risk of mortality was lowest at an eGFR of approximately 75 mL/min/1.73 m^2^ [15], and our study included older populations than those studies. Covariates for the models included age, sex, eGFR, BMI, blood pressure, presence of diabetes mellitus, current smoking status, and social support level. These were selected based on previous studies that identified the risk factors for life expectancy [18–23]. Statistical significance was set at *P* < 0.05.

An additional analysis of people who achieved the primary endpoint was conducted to assess their survival periods in unhealthy life years. The Kaplan–Meier method was used to estimate survival time by the baseline eGFR groups. Differences between the survival curves were tested using the Wilcoxon test. The test was used to measure differences between the two survival curves because a large proportion of events occur early [24, 25]. Statistical analyses were performed using Stata/MP software (version 17.0, Stata Corp MP, College Station, TX, USA).

## Results

### Baseline characteristics of participants

The data of 5,592 participants were analyzed (Additional file 3). The baseline characteristics are shown in Table 1. The eGFR ≥ 60 to < 75 group had the most participants. The average age was 66.8 years, and 43.5% were male. The eGFR < 45 group had older participants, with a mean age of 79.4 years. The eGFR groups had similar BMIs and systolic blood pressures. At baseline, 2.7% of participants were certified for social support (support level 2 or care level 1). Lower eGFR was associated with a higher proportion of participants receiving social support.

**Table 1:** Baseline characteristics of the study population

	eGFR (mL/min/1.73 m^2^)					
Variable	< 45 (*n* = 261)	≥ 45 to < 60 (*n* = 995)	≥ 60 to < 75 (*n* = 2,260)	≥ 75 to < 90 (*n* = 1,445)	≥ 90 (*n* = 631)	Overall (*n* = 5,592)
Age, years	79.4 (10.0)	71.7 (9.9)	67. 1 (9.5)	62.9 (10.3)	61.3 (10.5)	66.8 (10.9)
Sex, male	36.0%	48.6%	42.5%	45.4%	37.6%	43.5%
eGFR, mL/min/1.73 m^2^	35.7 (8.5)	54.1 (4.0)	67.8 (4.4)	81.0 (4.5)	99.5 (10.1)	70.8 (16.2)
Body mass index, kg/m^2^	23.4 (3.5)	23.6 (3.3)	23.3 (3.3)	22.9 (3.5)	23.1 (3.8)	23.2 (3.4)
Systolic blood pressure, mmHg	130 (18)	129 (17)	128 (17)	127 (17)	127 (17)	128 (17)
Diastolic blood pressure, mmHg	72 (11)	74 (11)	75 (11)	74 (11)	75 (11)	74 (11)
Diabetes mellitus	20.3%	16.2%	10.9%	11.6%	16.3%	13.1%
Current smoker	5.4%	11.0%	11.6%	16.9%	19.7%	13.5%
Social support level*						
Independent	83.9%	95.3%	98.4%	98.9%	98.4%	97.3%
Support level 1	2.7%	0.8%	0.1%	0.2%	0.0%	0.4%
Support level 2	2.3%	0.7%	0.4%	0.1%	0.5%	0.5%
Care level 1	11.1%	3.2%	1.1%	0.8%	1.1%	1.9%

Continuous variables are expressed as means (standard deviations). Categorical variables are expressed as proportions

Abbreviation: eGFR, estimated glomerular filtration rate

*Percentages may not total 100% because of rounding

### Primary endpoint by eGFR group

During the mean observation period of 6.4 years, 8.2% of the participants achieved the primary endpoint. The overall incidence rate of the primary endpoint was 13.0/1,000 person-years. Patients in the low eGFR group had a higher incidence rate of events than participants in the other groups (Additional file 4).

**Fig. 1:**
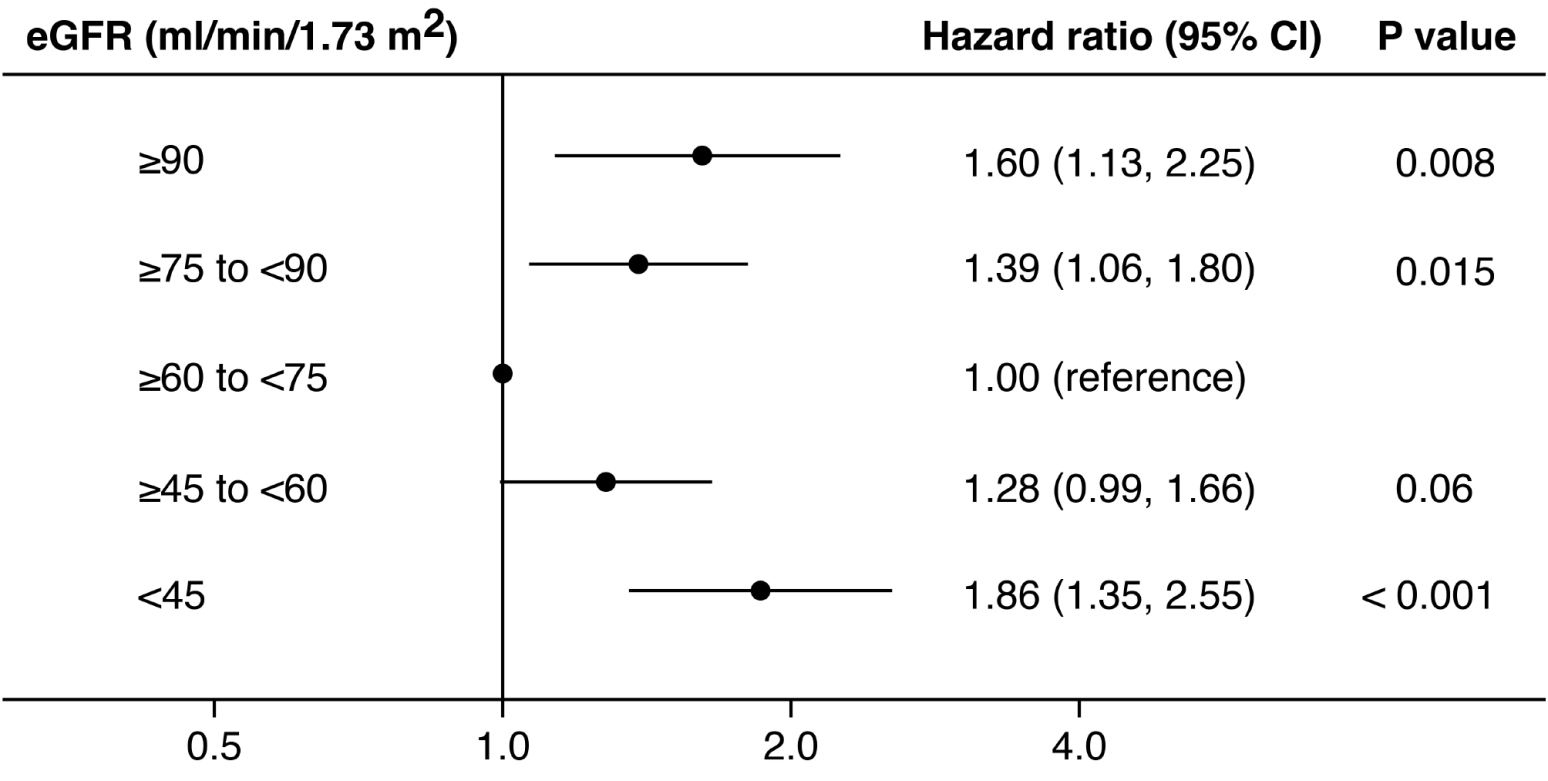
Hazard ratios for the risk of the primary endpoint by eGFR group The values are hazard ratios (95% confidence intervals) adjusted for age, sex, body mass index, systolic blood pressure, diabetes mellitus, current smoking status, and social support level Abbreviation: eGFR, estimated glomerular filtration rate

Table 2 shows the multivariable-adjusted risks for the primary endpoint according to the baseline eGFR. Model 1 was adjusted for age and sex, and model 2 additionally included BMI, systolic blood pressure, diabetes mellitus, and current smoker. The differences in hazard ratios (HRs) between the eGFR groups were statistically significant after adjusting for known factors in Models 1 and 2. These relationships remained significant, even after adjusting for baseline social support levels (Model 3). The HRs for the incidence of the primary endpoint were the lowest in the eGFR ≥ 60 to < 75 group, which was the reference group. Compared with the reference group, the lower eGFR group showed a higher HR trend (Fig. 1). In the eGFR < 45 group, the HR was 1.86 (95% confidence interval [CI]: 1.35 to 2.55). Similarly, HRs were higher in the higher eGFR groups compared with the reference group. In the eGFR ≥ 90 group, the HR was 1.60 (95% CI: 1.13 to 2.25). Additionally, the baseline social support level was associated with the primary endpoint (HR 5.25, 95% CI: 3.91 to 7.04).

**Table 2:** Hazard ratios for the risk of an unhealthy state by eGFR group

Variable	Model 1 HR (95% CI)	Model 2 HR (95% CI)	Model 3 HR (95% CI)
eGFR (ml/min/1.73 m^2^)			
≥ 90	1.71 (1.21, 2.40)	1.65 (1.17, 2.33)	1.60 (1.13, 2.25)
≥ 75 to < 90	1.38 (1.06, 1.79)	1.37 (1.05, 1.78)	1.39 (1.06, 1.80)
≥ 60 to < 75	1.00 (reference)	1.00 (reference)	1.00 (reference)
≥ 45 to < 60	1.32 (1.03, 1.71)	1.30 (1.01, 1.68)	1.28 (0.99, 1.66)
< 45	2.16 (1.57, 2.95)	2.11 (1.53, 2.89)	1.86 (1.35, 2.55)
Age (+ 5 years)	2.02 (1.90, 2.15)	2.04 (1.91, 2.18)	1.85 (1.73, 1.98)
Male (vs. female)	1.71 (1.42, 2.07)	1.55 (1.27, 1.89)	1.63 (1.34, 1.99)
Body mass index (+ 1 kg/m^2^)		1.00 (0.97, 1.03)	1.01 (0.98, 1.03)
Systolic blood pressure (+ 5 mmHg)		1.00 (0.97, 1.03)	1.00 (0.97, 1.03)
Diabetes mellitus (vs. no)		1.32 (1.04, 1.68)	1.26 (0.99, 1.60)
Current smoker (vs. no)		1.48 (1.11, 1.98)	1.52 (1.14, 2.03)
Social support level ^a^			5.25 (3.91, 7.04)

Values are presented as mean (95% confidence interval) changes in eGFR slopes adjusted for covariates

Abbreviations: eGFR, estimated glomerular filtration rate; HR, hazard ratio; vs., versus

^a^ Independence vs. support level 1, support level 2, and care level 1

### Annual costs of medical and long-term care by eGFR group

**Fig. 2:**
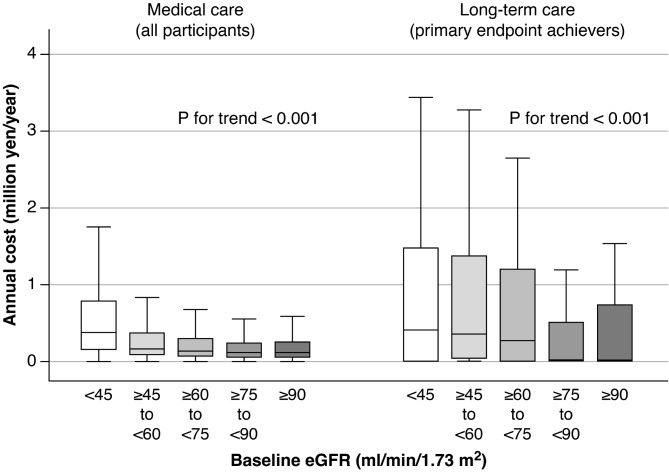
Annual medical and long-term care costs by eGFR group Annual costs of medical and long-term care were assessed for all participants (*N* = 5,592) and primary endpoint achievers (*N* = 461). The costs were calculated for each of the five eGFR groups Abbreviation: eGFR, estimated glomerular filtration rate

Regarding the secondary endpoint, the annual costs of medical and long-term care were assessed for all participants (*N* = 5,592), primary endpoint non-achievers (*N* = 5,131), and primary endpoint achievers (*N* = 461) (Additional file 5). For all participants, the annual cost of medical care was higher in the lower eGFR groups than in the higher eGFR groups (P for trend < 0.001). For the eGFR < 45 group, the median cost of medical care was 0.38 million yen/year (Fig. 2). For the primary endpoint achievers, the annual cost of long-term care was higher in the lower eGFR groups than in the higher eGFR groups (P for trend < 0.001). For the eGFR < 45 group, the median cost was 0.40 million yen/year.

### Unhealthy life years in the primary endpoint achievers

To assess long-term care costs for primary endpoint achievers (*N* = 461), a survival analysis was conducted (Additional file 6). In total, 52.3% of the participants died during the mean follow-up period of 1.2 years. The Kaplan–Meier curves showed significant differences among the five groups (*P* = 0.03). According to the curve, the lower eGFR groups (eGFR < 45 and ≥ 45 to < 60) had more unhealthy life years than the reference group (eGFR ≥ 60 to < 75).

## Discussion

Our study analyzed the association between kidney function and healthy life expectancy in community-dwelling adults, using data from the National Health Insurance System of Japan. Compared to eGFR ≥ 60 to < 75 mL/min/1.73 m^2^, both higher and lower eGFRs were associated with an increased risk of becoming unhealthy. Additionally, the lower eGFR groups had higher medical and long-term care costs than the higher eGFR groups did.

Our study showed that lower eGFR levels may be a prognostic factor of shorter healthy life expectancy. This result is consistent with those of previous studies, using other endpoints, such as disability adjusted life years (DALYs) [8, 26]. The risk magnitude is similar to that of delaying cancer treatment [27]. The risk may result from diseases related to kidney dysfunction, including cardiovascular and cerebrovascular disease, dementia, and fractures [7, 8, 28].

In addition to a lower eGFR, a higher eGFR was associated with a shorter healthy life expectancy. In our study participants, the high eGFR group had a higher prevalence of smoking and diabetes mellitus, suggesting less favorable lifestyle habits that may have influenced the results. Previous studies have shown a higher risk of death and cardiovascular disease events in the higher eGFR group. Although the effects of malignancy, lifestyle, and muscle mass have been suggested, the specific reasons for this are still unclear [29, 30]. It remains an important issue for future studies to understand the reasons for the reduced healthy life expectancy in the high eGFR group.

Additionally, the baseline social support levels were associated with shorter healthy life expectancy. Participants who received social support at baseline had a higher proportion of care level 1. This level refers to a condition in which partial care is needed for activities of daily living (ADL). Previous studies have reported that a decline in ADL can lead to further frailty [31]. This can make engaging in health-maintaining activities challenging, which may contribute to a further decline in their overall health status of individuals, regardless of their kidney function.

The lower eGFR groups had higher medical care costs than the higher eGFR groups did, consistent with the findings from previous studies [9–11]. The same trend was observed in the long-term care costs. When analyzing the subgroup that became unhealthy, these higher costs were from a longer unhealthy period in the lower eGFR groups. When analyzing the subgroup that became unhealthy, these higher costs may be attributed to a longer unhealthy period in the lower eGFR groups than in the higher eGFR groups. Thus, lower eGFR values are associated with health and economic burden.

### Strengths and limitations

Our study had several strengths. First, we focused on regions with large populations of older adults. This is important given the expected global aging of high-income countries. Second, our study used the National Health Insurance database, providing a detailed analysis of the association between kidney function and healthy life expectancy. Third, as the database contained both medical and long-term care data, the economic burden of healthcare insurance could be comprehensively analyzed.

This study had several limitations. First, it focused on the Japanese population. The study findings may not directly apply to other populations owing to differences in healthcare systems, lifestyles, and genetics. Second, this study targeted individuals who underwent health checkups. individuals with low health awareness, low socioeconomic status, or who live alone tend to avoid health checkups [32]. Therefore, the risk of a shortened healthy life expectancy may be underestimated. Third, we did not examine the diseases that caused kidney dysfunction, declined ADL, or death. Diseases such as polycystic kidney, chronic glomerulonephritis, cardiovascular diseases, or malignancies could have affected the results. Medications were also not considered. Therefore, the risk of a shortened healthy life expectancy may be inaccurate in either direction. Fourth, the classification of eGFR groups was based on a single creatinine measurement. It may not fully capture the dynamic nature of kidney function, because an individual’s eGFR classification can change over time. This limitation may have affected the analysis of long-term prognosis and led to uncertainty in the estimation of the associated risk.

## Conclusions

This study’s findings suggest that both high and low eGFRs are associated with an increased risk of an unhealthy state. Global initiatives will improve the prevention and treatment of CKD. Further investigation is warranted to determine whether these efforts may effectively reduce the future health and economic burdens.

## Electronic supplementary material

Below is the link to the electronic supplementary material.

Supplementary Material 1

## Data Availability

The data underlying this article will be shared on reasonable request from the corresponding author. There are ethical restrictions on public sharing of the anonymized dataset used in this study, primarily because of the potential identifiability of personal information within the data and materials. Access to confidential data may be granted to researchers who meet specific criteria determined by Kanazawa University. For inquiries regarding data access, please contact the designated representative of Kanazawa University.

## Abbreviations

CKD: Chronic kidney disease
KDB: Kokuho Database
BMI: Body mass index
KDIGO: Kidney Disease: Improving Global Outcomes
HRs: Hazard ratios
CI: Confidence interval
DALYs: Disability adjusted life years
BMI: Body mass index
eGFR: Estimated glomerular filtration rate
