# Supplementary material for: Relationship between kidney function and healthy life expectancy: A historical cohort study
Source: BMC Nephrol. 2025 Jan 13;26:21. doi: 10.1186/s12882-024-03843-0 (PMC11730782; doi:10.1186/s12882-024-03843-0)
Supplement: Supplementary file 1 — Supplementary Material 1 [file 12882_2024_3843_MOESM1_ESM.docx]

Additional Files

- File name: Additional file 1
- File format: .docx
- Title of data: Calculation method of the medical and long-term care costs
- Description of data: This file provides details on the cost components and calculation methods for the medical and long-term care costs used in the analysis.
- File name: Additional file 2
- File format: .docx
- Title of data: Definitions of social support levels in the Japanese long-term care system
- Description of data: There are eight levels of social support in Japanese long-term care insurance system. The levels depend on the degree of disability.
- File name: Additional file 3
- File format: .docx
- Title of data: Flow diagram of study participant selection
- Description of data: A total of 5,592 participants were included in the analysis, excluding those with missing covariates, missing date of birth and those with care level ≥2 at the time of enrollment.
- File name: Additional file 4
- File format: .docx
- Title of data: Incidence rate of events by eGFR group (primary endpoint)
- Description of data: Patients in the low eGFR group had a higher incidence rate of the primary endpoint than participants in the other groups.
- File name: Additional file 5
- File format: .docx
- Title of data: Annual medical care and long-term care costs by eGFR groups
- Description of data: Lower eGFRs may be associated with higher medical and long-term care costs.
- File name: Additional file 6
- File format: .docx
- Title of data: Unhealthy life years in primary endpoint achievers
- Description of data: The lower eGFR groups had more unhealthy life years than the higher eGFR groups did.

**Additional file 1.** Calculation method of the medical and long-term care costs

Japan has a universal health insurance system, and under this system, insured individuals receive medical and long-term care services at fees set by the government. Insured individuals pay 10–30% of the cost, depending on their age, while the insurer covers the rest. The costs in our analysis represent the total amount of services provided, which is the sum of payments by both Insured individuals and insurers. It does not include out-of-pocket spending for the non-covered services or transportation costs.

We calculated the annual costs of medical and long-term care for each participant using the following formula.

- C$i$: Total costs for participant $i$ (including the period after achieving the primary endpoint)
- T$i$: Total observation period for participant $i$ (including the period after achieving the primary endpoint)

The annual cost is calculated as follows:

$Annual cost for participant i = \frac{Ci (Total costs )}{Ti (Total observation period)}$)

This calculation was performed for all participants, both those who achieved the primary endpoint and those who did not. To perform this calculation, we used the dataset variables listed below [1].

**Medical care services**

1. Basic medical examination fee
   1. First visit fee
   2. Revisit fee
   3. Hospitalization fee
2. Specially listed medical service fee
   1. Guidance/management

Examples: Specified disease treatment guidance fee, including malignancy, dementia, lifestyle-related diseases, and pediatric diseases

- 1. In-home medical care

Example: Physician-nurse home visit fee

- 1. Tests

Example: Urinalysis, blood testing, microbiology, and interpretation of the results

- 1. Diagnostic imaging

Example: Fees for performing X-rays, computed tomography scans, magnetic resonance imaging scans, and nuclear medicine studies and interpreting the results

- 1. Medication

Examples: Drug fee, dispensation fee, and prescribing fee

- 1. Injection

Example: Intradermal, subcutaneous, or intramuscular injection

- 1. Rehabilitation

Examples: Rehabilitation for cardiovascular disease, cerebrovascular disease, and swallowing function

- 1. Specialized psychiatric therapy

Examples: Standard psychoanalysis therapy, cognitive behavioral therapy, and psychiatric care

- 1. Treatment

Examples: Wound treatment, chest drainage, arthrocentesis, and oxygen therapy

- 1. Surgery

Examples: Appendectomy, artificial joint replacement, and percutaneous coronary intervention

- 1. Anesthesia

Examples: Spinal anesthesia, general anesthesia, and nerve block treatment

- 1. Radiation therapy

Examples: Extracorporeal radiation, particle therapy, and gamma Knife radiosurgery

- 1. Pathology specimen preparation

Example: Cytology, immunostaining of pathological specimens, and pathological diagnosis

1. Inpatient dietetic treatment

**Long-term care services**

1. In-home Services
   1. Home-visit care (home help service)
   2. Home-visit bathing long-term service
   3. Home-visit nursing care
   4. Home-visit rehabilitation
   5. Guidance for management of in-home medical long-term care
   6. Outpatient day long-term care (day service)
   7. Outpatient rehabilitation (day care)
   8. Short-term admission for daily life long-term care (short stay)
   9. Short-term admission for recuperation
   10. Daily life long-term care admitted to a specified facility
   11. Rental service of equipment for long-term care covered by public aid
   12. Sales of specified equipment covered by public aid
2. Facility services
   1. Home-based care support services (Special nursing home for the older patients)
   2. Long-term care health facility
   3. Sanatorium medical facility for the older patients requiring long-term care
   4. Integrated facility for medical and long-term care
3. Community-based care services
   1. Regular visiting/on demand home-visit long-term/nursing care
   2. Home visit at night for long-term care
   3. Community-based outpatient day long-term care
   4. Outpatient long-term care of patients with dementia
   5. Multifunctional long-term care in a small group home
   6. Communal daily long-term care for patients with dementia (group home)
   7. Daily life long-term care for people admitted to a community-based specified facility
   8. Community-based facility for preventive daily long-term care of the older patient’s welfare instruments
   9. Combined Multiple Service (multifunctional long-term care in a small group home & home-visit nursing)
4. Preventive long-term care services
   1. Home-visit bathing service for preventive long-term care
   2. Home-visit nursing service for preventive long-term care
   3. Home-visit rehabilitation service for preventive long-term care
   4. Management and guidance for in-home medical service for preventive long-term care
   5. Outpatient rehabilitation service for preventive long-term care
   6. Short-term admission for daily preventive long-term care
   7. Short-term admission for recuperation for preventive long-term care
   8. Daily preventive long-term care admitted to a specified facility
   9. Sales of specific preventive long-term care welfare instruments
   10. Lending preventive long-term care welfare instruments
5. Community-based preventive long-term care services
   1. Outpatient care service for preventive long-term care for patients with dementia
   2. Multifunctional preventive long-term care in a small group home
   3. Daily life care service for preventive long-term care in communal living for patients with dementia
6. Comprehensive services for long-term care prevention/daily life support
   1. 1st house call services
   2. 1st day care services
   3. 1st living assistance1st care prevention support services
7. Others
   1. Allowance for home modification

## Additional file 2. Definitions of social support levels in the Japanese long-term care system

| **Social support level** | **Estimated total care minutes per day** * | **Expected individual condition** |
| --- | --- | --- |
| **Independent** | <25 | A person who can perform basic activities of daily living, such as walking and getting up, and instrumental activities of daily living, such as taking medications and using the telephone |
| **Support level 1** | ≥25 to <32 | A person who can perform basic and instrumental activities of daily living almost independently but who requires some assistance with instrumental activities of daily living to prevent progression to a care-dependent state |
| **Support level 2** | ≥32 to <50 | A person who can perform basic and instrumental activities of daily living almost independently, but who, because of a greater decline in physical function than those in support level 1, requires more support to prevent progression to a care-dependent state. |
| **Care level 1** | ≥32 to <50 | A person who almost is able to perform instrumental activities of daily living but continues to decline from the need for partial assistance to the need for partial care |
| **Care level 2** | ≥50 to <70 | A person who progresses from care level 1, requiring partial care not only with instrumental activities of daily living, but also with basic activities of daily living |
| **Care level 3** | ≥70 to <90 | A person who is substantially worse than level 2, requiring almost full care with both basic activities of daily living and instrumental activities of daily living |
| **Care level 4** | ≥90 to <110 | A person whose functional ability has declined further, making it difficult to perform any activities of daily living without care |
| **Care level 5** | ≥110 | A person with even greater functional decline than level 4, making it almost impossible to perform daily activities without care |

* To receive caregiving services, individuals or their families must apply to the local government office. After applying for caregiving, the city staff visit the home and perform an initial assessment, calculating the estimated total care minutes per day for activities such as grooming/bathing, eating, toileting, transferring, assisting with daily living, addressing behavioral issues, rehabilitation, and medical services. The Care Certification Review Committee determines the social support level using the initial assessment results and supporting documents, such as the primary physician's report [2, 3].

**
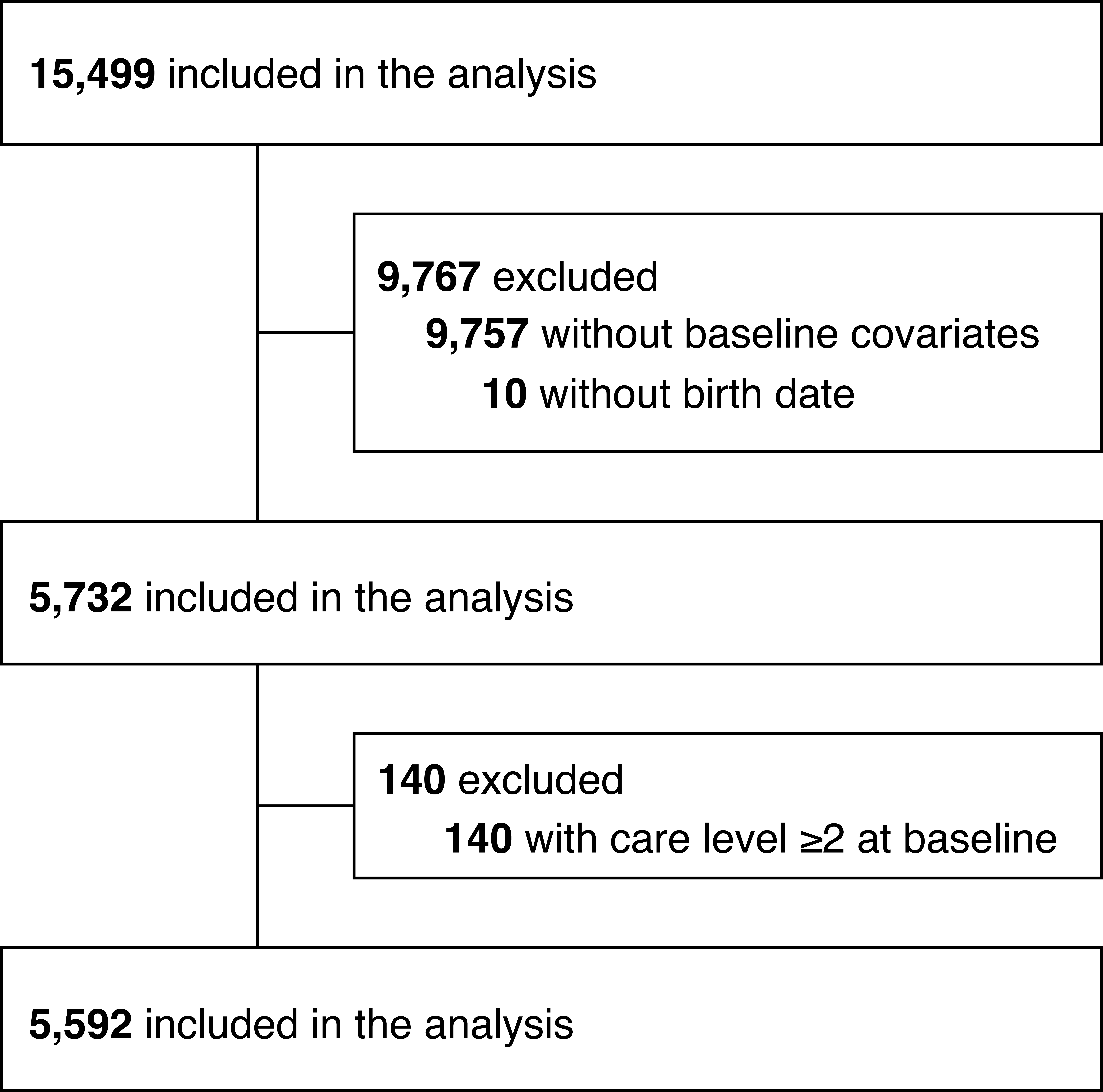
**

## Additional file 3. Flow diagram of study participant selection

## Additional file 4. Incidence rate of events by eGFR group (primary endpoint)

| eGFR  (mL/min/1.73 m^2^) | N |  | Incidence rate (per 1,000 PY) | | |
| --- | --- | --- | --- | --- | --- |
|  |  |  | Care level ≥2 | Death | Care level ≥2  or death |
| ≥90 | 631 |  | 4.4 | 5.3 | 9.6 |
| ≥75 to <90 | 1,445 |  | 4.8 | 4.5 | 9.4 |
| ≥60 to <75 | 2,260 |  | 5.9 | 3.9 | 9.8 |
| ≥45 to <60 | 995 |  | 14.1 | 6.8 | 20.9 |
| <45 | 261 |  | 48.8 | 25.5 | 74.2 |
| Total | 5,592 |  | 7.7 | 5.2 | 13.0 |

Abbreviation: PY, person year.





## Additional file 5. Annual medical care and long-term care costs by eGFR groups

Annual medical care costs (A) and annual long-term care costs (B) were assessed for all participants (N = 5,592), primary endpoint non-achievers (N = 5,131), and primary endpoint achievers (N = 461). Costs were calculated for each of the five eGFR groups.

eGFR, estimated glomerular filtration rate.

**

**

## Additional file 6. Unhealthy life years in primary endpoint achievers

Subgroup analysis focused on the primary endpoint participants (N = 461). Participants who reached the primary endpoint of death were considered to have died 1 day after the start of follow-up to account for the unhealthy period of those who experienced immediate death. eGFR, estimated glomerular filtration rate.

# References

1. Annual Health, Labour and Welfare Report 2021. <https://www.mhlw.go.jp/english/wp/wp-hw14/index.html> Accessed 15 October 2024.
2. Yamada M, Arai H. Long-term care system in Japan. Ann Geriatr Med Res. 2020;24:174-80. <https://doi.org/10.4235/agmr.20.0037>
3. Tsutsui T, Muramatsu N. Care-needs certification in the long-term care insurance system of Japan. J Am Geriatr Soc. 2005;53:522-27. <https://doi.org/10.1111/j.1532-5415.2005.53175.x>
